# The impact of emotional feedback in learning easy and difficult tasks – an ERP study

**DOI:** 10.3758/s13415-025-01284-2

**Published:** 2025-03-27

**Authors:** Jana Isabelle Braunwarth, Nicola Kristina Ferdinand

**Affiliations:** Department of Psychology, University of Wuppertal, Gaußstraße 20, 42119 Wuppertal, Germany

**Keywords:** Feedback processing, Feedback-related negativity, P3b, Emotionality, Working memory load

## Abstract

Learning from the emotional reaction of others is crucial in our everyday lives. We assumed that additional emotional information could be especially beneficial, when a task is difficult and the limits of working memory capacity are reached. For this reason, we examined whether a potential benefit of emotional feedback during reinforcement learning is dependent on working memory load. In addition to learning performance, we analysed the neural mechanisms of reinforcement learning by examining two event-related potentials (ERPs): feedback-related negativity (FRN) and P3b. Participants were divided into two difficulty groups (with *n* = 21 in the difficult and *n* = 22 in the easy group), performing a learning task with emotional or non-emotional feedback. Task difficulty was manipulated by varying the number of stimulus–response associations. Participants’ showed learning in all conditions. Emotional feedback led to increased accuracy and decreased reaction times in both groups. However, this benefit occurred earlier in the easy condition. The detection of unexpected events, as reflected in the peak-to-peak FRN, as well as working memory updating, as reflected in the P3b, were enhanced after emotional in contrast to non-emotional feedback for both groups. In contrast, task difficulty had no effect on the detection of unexpected events but led to a P3b that was more evenly distributed over the scalp, which could indicate that additional frontal resources were recruited to perform the difficult task. Our results suggest that working memory load and emotional information independently influence feedback processing without interacting.

**Supplementary Information:**

The online version contains supplementary material available at 10.3758/s13415-025-01284-2.

## Introduction

Adjusting our behavior in response to feedback is a fundamental aspect of our daily interactions. Feedback not only informs us about the correctness of our decisions, but also serves as a source of learning, allowing us to modify future actions. In an earlier study, emotional feedback led to better learning in older adults, whereas no benefit from emotional feedback was found in younger adults (Ferdinand & Hilz, 2020). This suggests that older adults were able to use the additional emotional information to compensate for age-related deficits, especially their decreased working memory capacity. This raises the question of whether younger adults’ feedback processing is, in general, not susceptible for emotional information or whether younger adults would also be able to benefit from emotional information when their working memory capacity is pushed to its limits. This study investigated feedback processing in a reinforcement learning paradigm under low and high working memory load in younger adults to get more insights on under which circumstances people benefit from emotional feedback and how this benefit is processed on a neural level by assessing two feedback-locked event-related potentials (ERPs): feedback-related negativity (FRN) and the P3b.

### Learning from feedback

During feedback-induced learning, distinct brain regions serve specific roles. The mediofrontal cortex, particularly the anterior cingulate cortex (ACC), and the mesencephalic dopamine system, are especially relevant for action monitoring (Cohen, 2008). Mesencephalic dopamine neurons show phasic increases for better-than-expected events and decreases for worse outcomes, serving as learning signals for the ACC (Alexander & Brown, 2011; Holroyd & Coles, 2002; Schultz, 2002). In the event-related potential, the FRN, a negative deflection peaking at approximately 250 ms after feedback presentation and primarily generated in the dorsal ACC, is found and reflects the detection of unexpected (negative) events (Ferdinand & Opitz, 2014; Gehring & Willoughby, 2002; Miltner et al., 1997; Yeung et al., 2004). There are different approaches to measure early feedback processing. The mean amplitude FRN has been found to be affected by valence, feedback probabilities, and magnitude (for a review see Martín, 2012; Gheza et al., 2018), with an enhanced negativity after negative feedback or an enhanced positivity after rewarding feedback, thus indicating a signed prediction error. Therefore, over the past years, the term reward positivity (RewP) has also been coined (Holroyd & Krigolson, 2007; Holroyd et al., 2008; Proudfit, 2015). In contrast, studies using a peak-to-peak measure of the FRN consistently found that it reflects the extent of the participant’s expectancy violation, i.e., the extent to which feedback is unpredicted by the participant. It has been demonstrated to be independent of the valence of the feedback and thereby may represent an unsigned prediction error (Ferdinand & Kray, 2013; Ferdinand et al., 2012; Oliveira et al., 2007; Pfabigan et al., 2011). This assumption is further strengthened by studies showing that the same expectancy manipulations that affect the peak-to-peak FRN also correlate with activation in the ACC (Ferdinand & Opitz, 2014). As we examine the contribution of expectancy violations to learning from feedback in this study, we will focus on the peak-to-peak FRN.

Following the detection of unexpected events, feedback undergoes further processing to lead to behavioral adaptation. According to the context updating theory, a P300 is elicited when an event is unexpected or novel, indicating the need for an update in the current cognitive context (Polich, 2007). The P300 is further subdivided into the P3a and P3b subcomponents, which differ in both function and topographical distribution. The P3a, which is typically observed over more frontal regions, is associated with the allocation of attentional resources to new stimuli. In contrast, the P3b, which displays a parieto-central topography, is linked to updating working memory with unexpected task-relevant information. The P3b is characterized by a positive deflection peaking between 300–500 ms post-feedback at parieto-central electrode sites, reflecting its role in integrating information into ongoing cognitive processing (Bellebaum & Daum, 2008; Ferdinand et al., 2012; Martín, 2012; Mecklinger et al., 1994; Polich, 2007; Ullsperger et al., 2014; Walentowska et al., 2016).

### Task difficulty and working memory load

Increasing task difficulty impacts learning, resulting in slower learning progress and diminished performance (Collins & Frank, 2012; Ferdinand, 2019; Gorlick et al., 2013; Luft, 2014). Consistent with the cognitive load theory, depending on the task, these are the results of a limited working memory capacity (Kirschner, 2002; Lin & Liang, 2021; Sweller, 1994). As a consequence, recent studies have explored how variations in task difficulty and their impact on working memory load influence feedback processing (Arbel, 2020; Ferdinand, 2019; Krigolson et al., 2015; Somon et al., 2019). For instance, Ferdinand (2019) investigated task difficulty in a probabilistic learning task with younger and older adults by manipulating the number of stimulus- response associations that had to be kept in working memory, and revealed slower learning in the more difficult condition. Similarly, Arbel (2020), explored task difficulty in a paired-association task with children, doubling the number of object-name pairs in the difficult condition and thereby increasing working memory load. They observed better performance in the easy as compared to the difficult task condition (for similar behavioral results, see Collins & Frank, 2012; Gorlick et al., 2013).

Results concerning the FRN are to date incongruent. For instance, Krigolson et al. (2015) investigated the neural mechanisms of reward processing by manipulating cognitive load in a time estimation task by adding a second task in the high cognitive load group. Despite similar behavioural task performance between groups, the high cognitive load group exhibited a less effective detection of unexpected events, reflected in a smaller peak FRN. The authors argued that competing resources result in a less functional mediofrontal reward system. Similar results were found by increasing the complexity of the feedback stimulus (Krigolson et al., 2012). In contrast, Ferdinand (2019) could not find an effect of task complexity on the peak-to-peak FRN in a probabilistic learning task, when the informational value of feedback stimuli was 100% valid. Also, Arbel (2020) and Somon et al. (2019) failed to find effects of working memory load on the FRN. Based on the latter studies, one would assume that even when working memory load is high, the early, automatic detection of prediction errors (reflected by the FRN) remains intact because this process relies on basic neural mechanisms that are not heavily influenced by the concurrent cognitive demands placed on working memory.

In contrast, ERP components like the P3b seem more strongly affected by working memory load, likely because they are involved in later stages of feedback processing, such as outcome evaluation and working memory updating, which are more reliant on attentional and working memory resources (Polich, 2007). Relatively few studies, however, investigated the influence of working memory load on the P3b during feedback-induced learning (Arbel, 2020; Ferdinand, 2019). Research examining the interaction between task complexity and feedback processing in reinforcement learning contexts has shown that under high working memory load, the ability to integrate feedback effectively can be impaired, leading to reduced P3b responses. For instance, Ferdinand (2019) found that the P3b after negative feedback was diminished in a task with a higher working memory load as compared to a task with a low load. Moreover, they found that the P3b was distributed more evenly over the scalp in the difficult task and concluded that additional frontal resources were recruited to perform the difficult task successfully (Cabeza et al., 2002; Ferdinand, 2019; Reuter-Lorenz et al., 2000; Tregellas et al., 2006). Arbel (2020) found that the P3b was less activated in children in the difficult task as well. Conversely, in an N-back task, working memory updating as indicated by the P3b was not sensitive to a higher cognitive load at all (Jia et al., 2022). Additionally, in a modified Flanker task, conducted by Somon et al. (2019), an increase in task difficulty by increasing the flanker’s task difficulty (with congruent and incongruent arrowheads) led to an enhanced P3b. The authors suggested that an enhanced insecurity led to a larger activation (i.e., needs more working memory updating). It should be noticed, however, that this task is no genuine learning paradigm and additionally puts participants under time pressure to provoke errors, both of which can fundamentally influence the cognitive processes involved.

In addition to reconciling inconsistent findings on the detrimental effects of increased working memory load—induced by an increased number of stimulus–response associations—on learning and feedback processing, the present study also aims to examine whether these detrimental effects can be mitigated by other relevant factors like socioemotional feedback.

### Socioemotional feedback

Feedback with socioemotional information is pervasive in daily life and considered particularly relevant and motivating (Adolphs, 2009; Rolls, 2000; Ruff & Fehr, 2014). Thus, numerous studies have explored social dynamics and their impact on performance (Boksem et al., 2011a, 2011b; Koban & Pourtois, 2014; Rak et al., 2013; Thoma & Bellebaum, 2012; Van Meel & Van Heijningen, 2010), whereas others investigated how facial stimuli influence feedback processing (Dekkers et al., 2015; Ferdinand & Hilz, 2020; Hurlemann et al., 2010; Pfabigan et al., 2014; Schulreich et al., 2013; Stavropoulos & Carver, 2014).

Hurlemann et al. (2010), for instance, used social (happy vs. angry faces) and nonsocial (red vs. green light) feedback in a paired-association task and found better performance after social feedback. Pfabigan et al. (2014) used a time estimation task to investigate whether social (happy vs. angry faces) compared with nonsocial (+ vs. −) feedback would strengthen feedback processing. However, they found no influence of feedback type on the peak-to-peak FRN, possibly due to perceptual differences in stimulus complexity (Liu & Gehring, 2009; Matyjek et al., 2020; Pfabigan et al., 2015). To address this, Pfabigan et al. (2019) developed similar social (thumps up vs. thumps down) and nonsocial (+ vs. −), complex and noncomplex stimuli and found an enlarged peak-to-peak FRN for social stimuli in a similar task. Nevertheless, time estimation tasks are not designed to show learning over time, thus conclusions concerning a possible learning benefit for social feedback are rather limited. Therefore, Sailer et al. (2023) used a probabilistic reward learning task but did not find an advantage of social over nonsocial feedback. In contrast, Ferdinand and Hilz (2020) used a probabilistic learning task with emotional (happy vs. disgusted) or non-emotional (neutral) face feedback to investigate learning across age groups and found enhanced learning from emotional feedback in older adults. Neutral faces, however, elicited larger peak-to-peak FRNs than emotional faces, most probably due to their unexpectedness and maybe even negative connotation in everyday communication.

As for working memory updating, some studies have found that participants exhibited larger P3b amplitudes after emotional stimuli, indicating stronger updating processes due to heightened feedback relevance (Pfabigan et al., 2014, 2019; Schulreich et al., 2013). However, Ferdinand and Hilz (2020) found that older adults benefited from emotional feedback during learning, whereas younger adults showed no such effect. This suggests that older adults were able to use the additional emotional information to compensate for age-related deficits during learning from feedback, which could be mainly due to their decreased working memory capacity. This raises the question of whether younger adults’ feedback processing is, in general, not susceptible for emotional information or whether younger adults would also be able to benefit from emotional information when their working memory capacity is pushed to its limits.

Taken together, the results of studies on feedback processing with socioemotional information are inconsistent. This is partly the result of methodological challenges in creating perceptually comparable conditions. Also, working memory load has not explicitly been investigated in this context, so differences due to different working memory load of the tasks or between participants cannot be excluded.

### The present study

This study was designed to explore whether a potential benefit of emotional feedback during learning and feedback processing is dependent on working memory load in younger adults. We conducted a probabilistic learning task that included emotional and non-emotional feedback and manipulated working memory load by varying the amount of to-be-learned stimulus–response associations. Additionally, we carefully controlled the feedback stimuli for visual complexity and thus used similarly complex emotional and non-emotional feedback stimuli by scrambling faces for the non-emotional condition. With this approach, we aimed to ensure that only one condition contained emotional information.

We assumed that young participants would benefit from emotional feedback in more difficult tasks when reaching their processing limit, i.e., when working memory load is high. This should be visible in learning performance, i.e., reduced reaction times and increased accuracy. Emotional feedback should also lead to strengthened feedback processing. Specifically, emotional feedback should be followed by an enhanced detection of unexpected events in the difficult task as reflected by a larger peak-to-peak FRN as well as enhanced working memory updating as indicated by an enlarged P3b. Lastly, we expected a frontal shift of the P3b in the difficult group, indexing the recruitment of additional frontal resources to successfully perform the more difficult task.

## Methods

### Participants

A priori conducted power analysis with a power of 0.8, effect size of 0.15 and $\alpha$= 0.05 revealed the need for 18 participants for each group. To account for possible drop-outs, we recruited 25 younger adults between 19 and 29 years for each group. Inclusion criteria were right handedness, no psychiatric or neurological diseases, and normal or corrected-to-normal vision. Three participants were excluded due to excessive EEG artifacts, one for left-handedness, another for insufficient learning performance (in no learning quarter and feedback condition accuracy above chance level), and two for insufficient number of negative trials (< 20). The final sample consisted of 22 participants in the easy group (mean age = 22.5 years, *SD* = 3.05; 12 women) and 21 participants in the difficult group (mean age = 22.9 years, *SD* = 2.46; 10 women). Participants were recruited through social media and university-specific websites and either received course credit or 25€ expense allowance. We followed the Declaration of Helsinki. The study was approved by the ethics committee of the University of Saarland, and this positive vote was acknowledged by the ethics board of the University of Wuppertal after transferring the project. All participants signed informed consent before participation.

### Stimuli

In the present study, participants completed a probabilistic learning task in four learning blocks (adapted from Ferdinand & Hilz, 2020), in which they learned a stimulus–response association by pressing a button followed by feedback. The stimuli consisted of different object groups, such as furniture and were taken from a standardized object database (Snodgrass & Vanderwart, 1980). Each object had a size of 281 × 197 or 197 × 281 pixel. Every learning block comprised six objects, with three objects connected to emotional and three objects connected to non-emotional feedback, counterbalanced across participants. The feedback stimuli were taken from the FACES database (Ebner et al., 2010) and had a pixel size of 161 × 201. Analogous to the study by Ferdinand and Hilz (2020), the emotional feedback condition consisted of happy faces for positive and disgusted faces for negative feedback. The non-emotional feedback condition consisted of neutral faces of the same database. However, neutral faces might still provoke negative emotional reactions (Ferdinand & Hilz, 2020). Therefore, because equally complex feedback stimuli were needed for the emotional and non-emotional condition, the neutral faces were scrambled to create non-emotional stimuli that were similarly complex to emotional faces without emotionality (Liu et al., 2014; Pfabigan et al., 2015; Shigeto et al., 2011) but still contain parts of a face as research has shown that different brain areas are activated during face vs. object processing (Itier & Taylor, 2004; McCarthy et al., 1997; Sergent & Signoret, 1992). The stimuli were scrambled using the scramble function of webmorph.org (DeBruine, 2018) with a size of 14 × 14 mm for each scramble. To address age and gender biases, four faces (young woman, old woman, young man, old man) were counterbalanced across participants leading to participants seeing only one of the four faces as feedback stimulus, but all four faces being used equally often in the experiment.

**Fig. 1:**
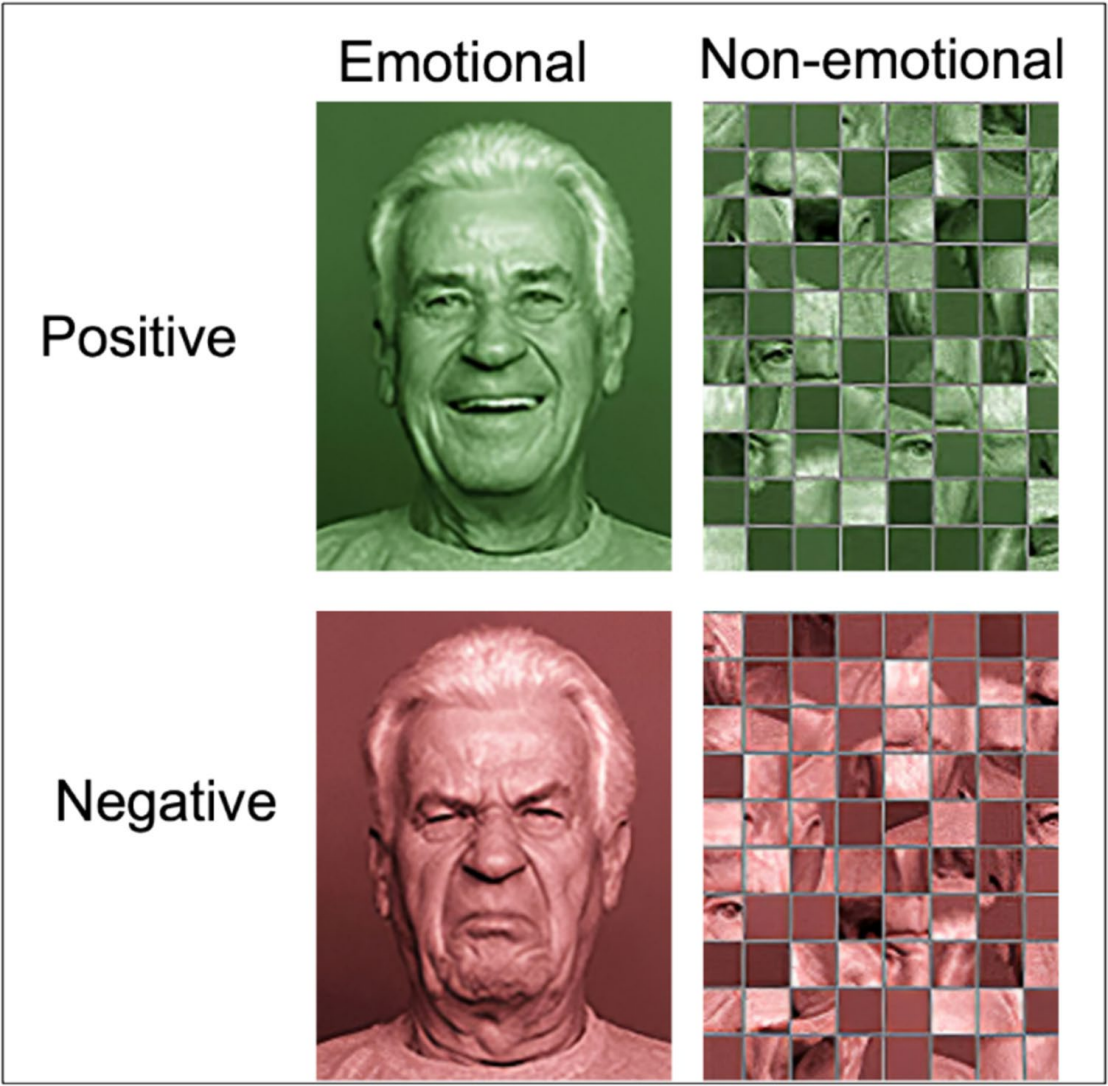
Emotional and non-emotional positive and negative feedback stimuli

To mark correct vs. incorrect response feedback, emotional and non-emotional feedback stimuli were coloured in green for positive and red for negative feedback. These colours intuitively indicate right or wrong as do the emotions in the emotional condition and should be helpful for discriminating between positive and negative feedback (Fig. 1). All images appeared on a grayscale background.

### Task

**Fig. 2:**
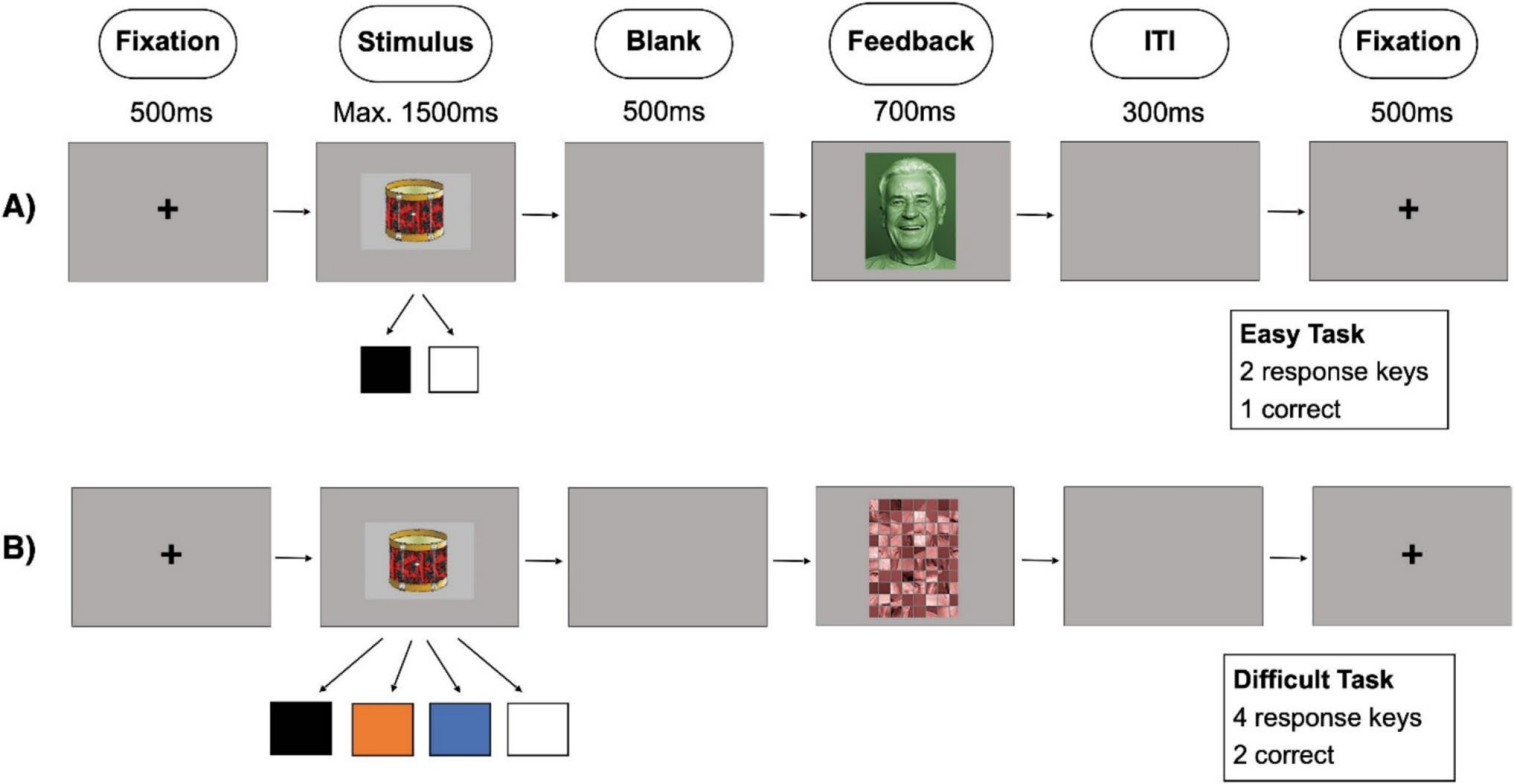
Exemplary trial procedure and timing for easy (**A**) and difficult (**B**) tasks with (**A**) presenting emotional and (**B**) presenting non-emotional feedback

Participants in the easy group were instructed to act as moving helpers, deciding whether objects belonged in the black or white truck. They used coloured response keys (“c” for black, “m” for white) for item allocation and received feedback from their boss on their choices (Fig. 2). Participants in the difficult group performed a similar task but had to allocate objects into two of four trucks using different coloured keys (“c” for black, “v” for orange, “n” for blue, “m” for white). With this approach, we aimed at increasing the load on working memory by doubling the number of associations to learn. Specifically, participants first had to find the one correct key, memorize it, and additionally find the second correct key out of the above mentioned four keys. For instance, in one of the learning blocks, participants were tasked with allocating items from the category “fruits.” They had to determine that the black and white trucks were the correct choices for the trials in which the apple appeared, whereas the blue and orange trucks were the appropriate selections in the trials, showing the banana. In every trial, only one response could be given. In the difficult condition, this meant that across multiple trials with the same object—mostly with other trials in between—participants needed to learn both correct responses. After each block, participants in both groups completed a brief retrieval task on object assignments, receiving feedback on their accuracy at the end. For this, every object was shown once, and participants had to press the correct association button. In the difficult group, it was emphasized that pressing the same button twice for an object was not allowed. This was done to ensure that participants in the difficult condition actually tried to learn two out of four correct responses for each object.

The probability of receiving valid feedback was 90%. Ten percent of the presented feedback was invalid, so one-trial learning could be prevented (Eppinger et al., 2008; Ferdinand & Hilz, 2020; Holroyd & Coles, 2002). Participants were informed about the possibility of receiving invalid feedback. It was explained that, due to the supervisor’s high workload, not all allocations were reviewed accurately, which in rare cases could lead to negative feedback even though the participants' allocations had been correct, and vice versa. However, it was emphasized that such instances of invalid feedback would occur only rarely. Response time was individually adapted (600–1500 ms) based on participants’ performance. With this approach, we aimed at minimizing individual differences within a group mainly concerning the timeouts. The experiment started with 1000 ms in the first trial. In case of exceeding this time, participants received a message on the screen stating “zu langsam” (German for “too slow”). Participants within the specified time range for 20 trials received a 100-ms reduction for the subsequent 20 trials. If they exceeded the limit once, the time range remained unchanged for the next 20 trials. Exceeding the time window more than once resulted in a 100-ms increase for the next 20 trials.

The experiment consisted of 720 trials divided into four learning blocks, each containing 180 trials. Each block was divided into four learning quarters for analyses. All participants practiced eight trials of the easy condition with the option to repeat if desired. The difficult group performed 90 trials of the easy condition to assess whether there were preexisting differences between the groups and to ensure a more comparable analysis across conditions. Short breaks with flexible duration were allowed every 60 trials.

### Procedure

After the participant arrived, the consent form was signed. To assess handedness, participants were asked to fill out the Edinburgh Handedness Questionnaire (Oldfield, 1971), followed by a paper–pencil version of the digit symbol substitution test (DSST) (Jaeger, 2018). Moreover, they completed a demographic survey, addressing extra aspects, such as exercise, nutrition, and social participation. Further questionnaires included the Emotion Regulation Questionnaire (Abler & Kessler, 2011) and the German version of the Interpersonal Reactivity Index (IRI) (Paulus, 2009). We used the IRI as empathy has been shown to affect social feedback processing (Van Meel & Van Heijingen, 2010; Albrecht & Bellebaum, 2021). Additionally, participants performed a digital version of the Mehrfachwahl-Wortschatz-Intelligenztest (MWT-B; Lehrl, 2005). Participants then were prepared for EEG recording and started the probabilistic learning task. They were seated in an electrically shielded and sound-proof EEG cabin in front of a 27-in screen within a distance of 60 cm. After the experiment, participants were asked to complete follow-up questions regarding their last night’s sleep, concentration, and strategies used during learning. They then received expense allowance and left.

### EEG recording and analysis

EEG was recorded using BrainVision Recorder (Version 1.23.0001, Brain Products GmbH, Gilching, Germany) and the paradigm was presented by E-Prime 3.0 (Psychology Software Tools, Pittsburgh, PA). A total of 58 active silver/silver chloride electrodes were attached according to the international 10–20 system (Jasper, 1958) to an elastic electrode cap. The ground electrode was placed at position AFz and the left mastoid served as online reference. An electrooculogram (EOG) was recorded for offline eye movement correction. Therefore, electrodes were placed supra- and infraorbitally to the right eye and near the outer canthi of both eyes. All impedances were kept below k $\Omega$ 20. EEG and EOG were filtered online by using a low-pass filter (250 Hz) and digitized with a sampling rate of 500 Hz. Data were preprocessed by using Matlab R2017b (The Mathworks Inc., Natick, MA) and the eeglab toolbox v.2021.1 (Delorme & Makeig, 2004). The EEG data were downsampled to 250 Hz, and filtered offline with a high-pass filter of 0.01 Hz and a low-pass filter of 30 Hz. To balance the signal across hemispheres, data were re-referenced offline to the average of the two mastoids. An independent component analysis was performed to extract eye movement-related components from the data. We then used IClabel to flag those artifacts, additionally checked those manually, and discarded eye movement-related components from the data (Pion-Tonachini et al., 2019). Afterwards, epochs were averaged for each subject and condition for the relevant segments (emotional positive, emotional negative, non-emotional positive, non-emotional negative feedback) and were cut using a time window of 900 ms, including a prestimulus baseline of − 100 ms using ERPlab v8.30 (Lopez-Calderon & Luck, 2014). Lastly, epochs exceeding 75 µV were removed in an additional artefact rejection step.

Based on previous literature, we decided to calculate the peak-to-peak FRN by subtracting the negativity of a time window of 240 ms to 340 ms from the preceding positivity in a time window of 180 ms to 240 ms postemotional feedback presentation at channel FCz (Ferdinand & Hilz, 2020; Ferdinand et al., 2012; Holroyd et al., 2006). After visual inspection, however, we found that this time window was not suitable for the non-emotional condition, because too many peaks were not adequately captured. A possible reason for this could be that the scrambled stimuli led to a prolonged FRN because of a higher complexity (Pfabigan et al., 2019). We then decided to use a collapsed localizer approach (Luck & Gaspelin, 2017) and used a time window of 180–300 ms to determine the P2 peak and of 240–370 ms to determine the N2 peak for both groups and all conditions at channel FCz. The P3b was measured from the mean value between 350 and 550 ms. This time window was taken based on previous studies (Martín, 2012) and after visual inspection. To be able to examine difficulty related frontal shifts, the P3b was analysed at three midline electrodes: Fz, Cz, and Pz.

### Data analysis

We employed a between-subjects design to minimize potential exhaustion effects among participants, which could arise from the substantial number of trials required for robust ERP analyses (Boudewyn et al., 2018). Additionally, we controlled for fluid and crystallized intelligence between groups and conducted a baseline analysis to assess any differences between the groups to make a comparison more feasible. Thus, we conducted mixed repeated measures ANOVAs for accuracy and reaction times, with Task Difficulty (easy, difficult) as a between-subject factor and Feedback Condition (emotional, non-emotional) and Learning Quarter (1,2,3,4) as within-subject factors. Trials with reaction times < 100 ms were excluded from behavioral analyses. Additionally, incorrect trials were excluded from the reaction time analyses. In case of significant main effects and interactions including the factor Learning Quarter, only pairwise comparisons for Learning Quarter 1 vs. Learning Quarter 2, Learning Quarter 2 vs. Learning Quarter 3, and Learning Quarter 3 vs. Learning Quarter 4 will be reported to reduce the number of comparisons. The Peak-to-peak FRN was analysed using a mixed-repeated measures ANOVA with Task Difficulty as a between-subject factor and Feedback Condition (emotional, non-emotional) and Valence (positive, negative) as within-subject factors at channel FCz. Mean P3b analysis employed a mixed-repeated measures ANOVA with Task Difficulty as a between-subject factor, and Feedback Condition (emotional, non-emotional), Valence (positive, negative), and Channel (Fz, Cz, Pz) as within-subject factors. Because we assume topography differences, planned contrasts comparing Fz vs. Cz and Cz vs. Pz were included. Because there is evidence that ERPs change over time and during learning, we included an additional control factor Learning Half (first, second) in a further ANOVA (Eppinger et al., 2008; Holroyd & Coles, 2002; Wurm et al., 2020). However, because a change in ERPs over time was not part of our hypotheses, we report those effects in the supplementary material.

Greenhouse–Geisser correction was applied for sphericity violations, and Bonferroni correction was used for post-hoc testing. If the assumption of sphericity was violated, the Greenhouse–Geisser correction was applied to both behavioral and ERP data. The adjusted *p*-values, and uncorrected degrees of freedom, are reported. Significance level was set to $\alpha$= 0.05. All analyses were performed in R Studio Version 3.6.3 (R Core Team, 2020).

## Results

### General group differences

To control for baseline group differences and differences in confounding variables, performance in the easy baseline trials as well as in the measures for fluid and crystallized intelligence were compared. The ANOVA with the between-subject factor Task Difficulty (easy, difficult) and the within-subject factor Feedback Condition (emotional, non-emotional) did not reveal any differences in reaction times or accuracy and no interactions with Feedback Condition (all *p* values > 0.29). Possible group differences for fluid and crystallized intelligence were analysed by using *t*-tests and revealed no effects in the MWT-B or DSST (all *p* values > 0.47). Lastly, we analysed empathy scores between groups by using *t*-tests, resulting in no significant differences (all *p* values > 0.32).

### Accuracy

The mixed repeated measures ANOVA with the between-participants’ factor Task Difficulty (easy, difficult) and the within-participants’ factors Feedback Condition (emotional, non-emotional) and Learning Quarter (Q1, Q2, Q3, Q4) resulted in a main effect for Feedback Condition (*F*(1,41) = 5.09, *p* = 0.029, ${\eta }_{p}^{2}$ = 0.11), revealing that emotional feedback led to higher accuracies than non-emotional feedback. Furthermore, significant main effects were found for Task Difficulty (*F*(1,41) = 29.09, *p* < 0.001, ${\eta }_{p}^{2}$ = 0.42) and Learning Quarter (*F*(1,41) = 135.04, *p* < 0.001, ${\eta }_{p}^{2}$ = 0.77), as well as an interaction between Task Difficulty and Learning Quarter (*F*(3,123) = 3.42, *p* = 0.029, ${\eta }_{p}^{2}$ = 0.08). Post-hoc tests showed that accuracy was worse in the difficult than the easy group in all four learning quarters (Q1: *t(*75.9) = − 5.81, *p* < 0.001); Q2: *t*(69.7) = − 6.51, *p* < 0.001); Q3: *t*(57.3) = − 5.69, *p* < 0.001, Q4: *t*(57.7) = − 5.01,* p* < 0.001). In the difficult group, accuracies increased from Learning Quarter 1 to 2 (*t*(41) = − 5.38, *p* < 0.001), Learning Quarter 2 to 3 (*t*(41) = − 5.74, *p* < 0.001), and Learning Quarter 3 to 4 (*t*(41) = − 3.66, *p* = 0.004). For the easy group, accuracies also increased from Learning Quarter 1 to 2 (*t*(43) = − 13.89, *p* < 0.001) and Learning Quarter 2 to 3 (*t*(43) = − 4.53, *p* < 0.001), whereas no significant change in accuracy was found from Learning Quarter 3 to 4 (*p* = 0.371). No significant interactions including the factors Feedback Condition and Task Difficulty were found (all *p* values > 0.080).

### Reaction times

To analyse reaction times, we conducted a mixed repeated measures ANOVA, analogous to the one for assessing accuracy. A main effect for Task Difficulty (*F*(1,41) = 6.08, *p* = 0.018, ${\eta }_{p}^{2}$ = 0.13), indicating faster reaction times in the easy than the difficult condition, and a main effect of Learning Quarter (*F*(3,123) = 24.71, *p* < 0.001, ${\eta }_{p}^{2}$ = 0.37), showing decreasing reaction times, were found. In addition, a main effect for Feedback Condition (*F*(1,41) = 7.78, *p* = 0.008, ${\eta }_{p}^{2}$ = 0.16) and interactions between Learning Quarter and Feedback Condition (*F*(3,123) = 3.26, *p* = 0.024, ${\eta }_{p}^{2}$ = 0.07) and between Task Difficulty, Learning Quarter and Feedback Condition were revealed (*F*(3,123) = 3.08, *p* = 0.030, ${\eta }_{p}^{2}$ = 0.07).

**Fig. 3:**
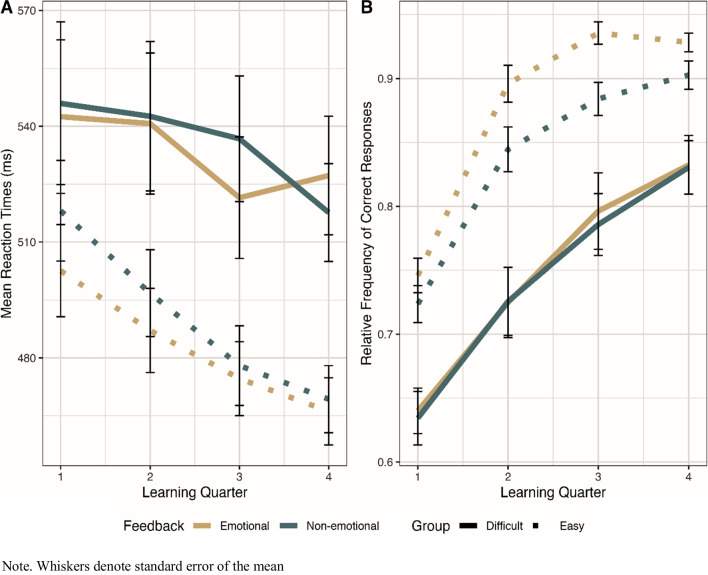
Mean reaction times (**A**) and accuracy across learning quarters (**B**) for both difficulty groups and feedback conditions

To resolve these interactions, we conducted ANOVAs separately for each group (Fig. 3). The analysis for the easy group resulted in main effects for Feedback Condition (*F*(1,21) = 8.70, *p* = 0.008, ${\eta }_{p}^{2}$ = 0.29), Learning Quarter (*F*(3,63) = 30.2, *p* < 0.001, ${\eta }_{p}^{2}$ = 0.59), and their interaction (*F*(3,63) = 2.83, *p* = 0.046, ${\eta }_{p}^{2}$ = 0.12). Post-hoc *t*-tests showed decreasing reaction times over learning quarters after emotional (Q1 vs. Q2 (*t*(21) = 3.42, *p* = 0.015), Q2 vs. Q3 (*t*(21) = 3.15, *p* = 0.029), Q3 vs. Q4 (*t*(21) = 3.14,* p* = 0.03) as well as after non-emotional feedback (Q1 vs. Q2 (*t*(21) = 3.27, *p* = 0.022), Q2 vs. Q3 (*t*(21) = 4.84, *p* < 0.001), Q3 vs. Q4 (*t*(21) = 3.16, *p* = 0.029). Additionally, participants in the easy group had faster reaction times after emotional than non-emotional feedback in Learning Quarters 1 (*t*(21) = − 2.76, *p* = 0.012) and 2 (*t*(21) = − 2.39, *p* = 0.026), whereas no such effect was found for Learning Quarters 3 and 4 (all *p* values > 0.26).

The analysis for the difficult group revealed a main effect for Learning Quarter (*F*(3,60) = 4.79, *p* = 0.025, ${\eta }_{p}^{2}$ = 0.02) and an interaction between Feedback Condition and Learning Quarter (*F*(3,60) = 3.22, *p* = 0.029, ${\eta }_{p}^{2}$ = 0.14). Post-hoc *t*-tests revealed shorter reaction times after emotional than non-emotional feedback in Learning Quarter 3 only (*t*(20) = − 2.85, *p* = 0.01). After emotional feedback, reaction times significantly decreased from Learning Quarter 2 to 3 only (*t*(20) = 3.33, *p* = 0.02), whereas no significant differences were found between Learning Quarter 1 vs. 2 (*p* = 1) and Learning Quarter 3 vs. 4 (*p* = 1). After non-emotional feedback, reaction times significantly decreased between Learning Quarter 3 vs. 4 (*t*(20) = 3.66, *p* = 0.009), whereas no such effects were found between Learning Quarter 1 vs. 2 (*p* = 1) and Learning Quarter 2 vs. 3 (*p* = 1).

### Peak-to-Peak FRN

**Fig. 4:**
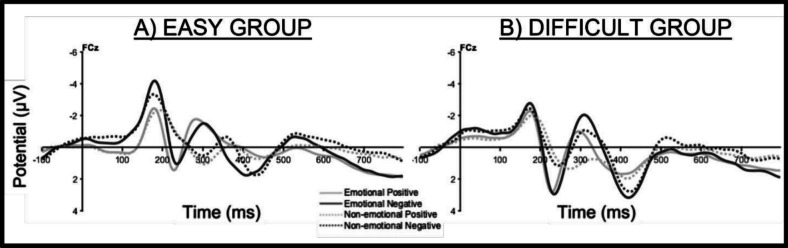
Feedback-locked ERP waveforms at Channel FCz for the easy (**A**) and difficult (**B**) groups

**Fig. 5:**
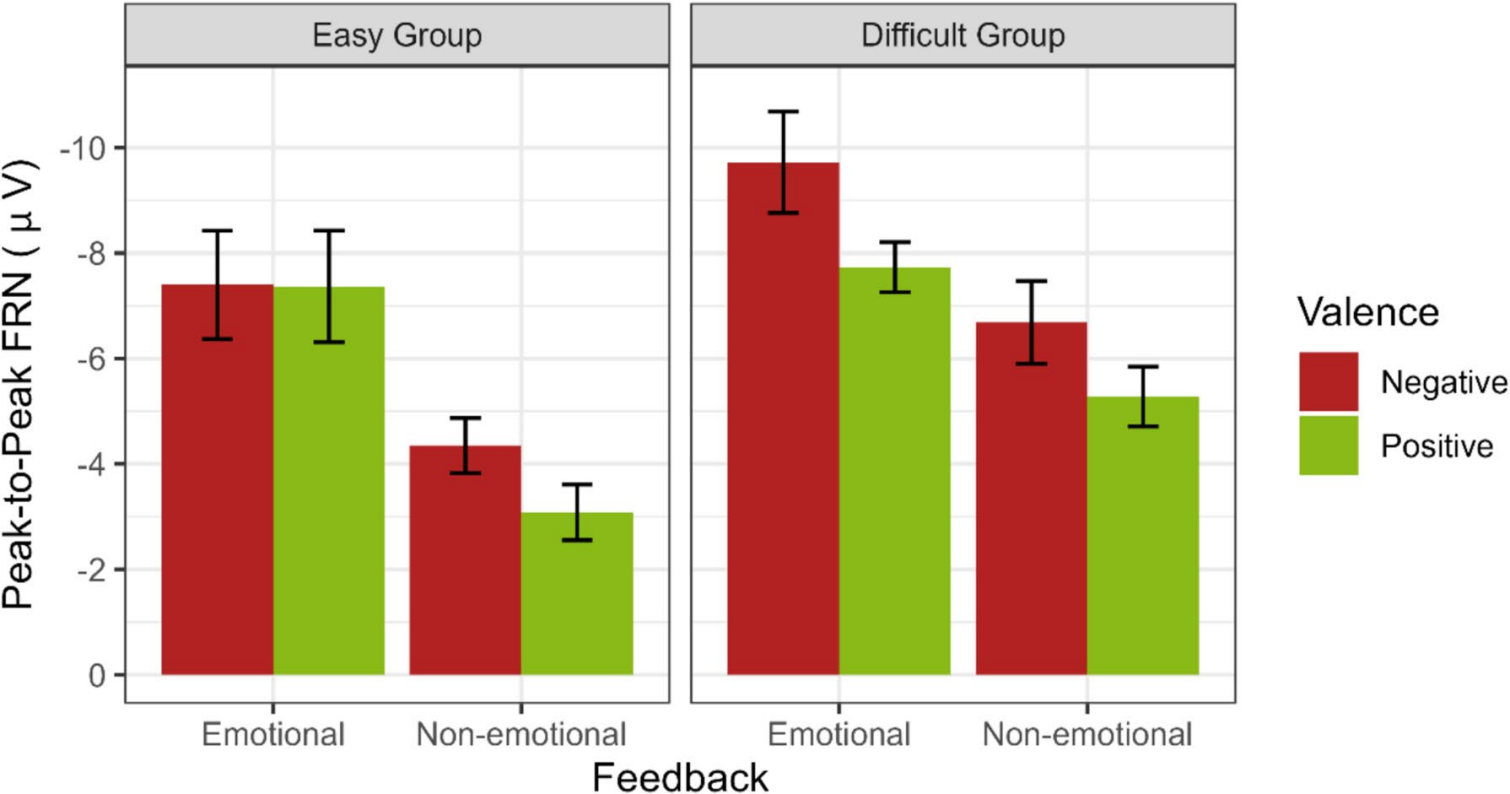
Peak-to-peak FRN at channel FCz for both groups and emotional and non-emotional feedback

The ANOVA with the factors Task Difficulty (easy, difficult), Feedback Condition (emotional, non-emotional), and Valence (positive, negative) on the peak-to-peak FRN at electrode FCz (Fig. 4) revealed a main effect for Feedback Condition (*F*(1,41) = 57.56, *p* < 0.001, ${\eta }_{p}^{2}$ = 0.58), showing a larger peak-to-peak FRN after emotional than non-emotional feedback. Additionally, a main effect for Valence (*F*(1,41) = 11.47, *p* = 0.002, ${\eta }_{p}^{2}$ = 0.22) was found, reflecting a larger peak-to-peak FRN after negative than positive feedback. No main effect of Task Difficulty (*p* = 0.054) nor a significant interaction with Task Difficulty was revealed (all *p* values ≥ 0.13) (Fig. 5).

### P3b

**Fig. 6:**
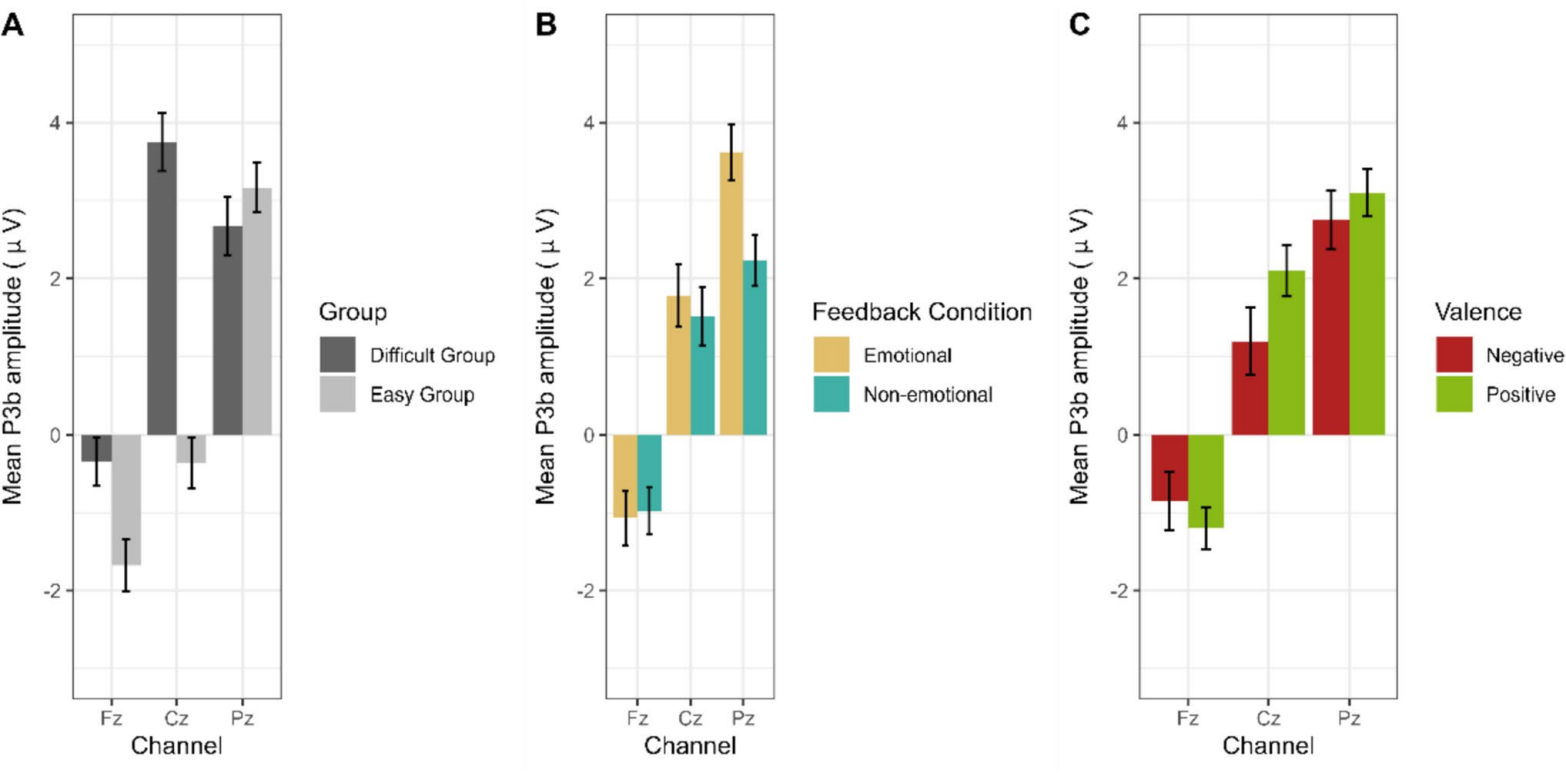
Mean P3b ANOVA two-way interactions. **A**) Task Difficulty x Channel; **B**) Feedback Condition x Channel; **C**) Valence x Channel

The ANOVA with the factors Task Difficulty (easy, difficult), Feedback Condition (emotional, non-emotional), Valence (positive, negative), and Channel (Fz, Cz, Pz) on mean amplitude P3b identified significant main effects for Feedback Condition (*F*(1,41) = 5.39, *p* = 0.025, ${\eta }_{p}^{2}$ = 0.12) and Channel (*F*(2,82) = 22.19, *p* < 0.001, ${\eta }_{p}^{2}$ = 0.35. In addition, two-way interactions between Task Difficulty and Channel (*F*(2,82) = 7.39, *p* = 0.003, ${\eta }_{p}^{2}$ = 0.15), Valence and Channel (*F*(2,82) = 5.58, *p* = 0.005, ${\eta }_{p}^{2}$=0.12), and Feedback Condition and Channel (*F*(2,82) = 11.45, *p* < 0.001, ${\eta }_{p}^{2}$=0.22) emerged (Fig. 6).

The analysis of the interaction between Task Difficulty and Channel (Fig. 6A) revealed significant amplitude differences across electrode sites. In the easy condition, the P3b had a clearly parietal topography as its amplitude was larger at Pz vs. Cz (*t*(175) = − 10.2, *p* < 0.001) and larger at Cz vs. Fz (*t*(175) = − 5.00, *p* < 0.001). In the difficult condition, P3b amplitudes were larger at Cz vs. Fz (*t*(167) = − 12.7, *p* < 0.001) and larger at Cz vs. Pz (*t*(167) = 2.4, *p* = 0.045), revealing a central P3b maximum. Also, the P3b was larger in the difficult than the easy group at electrode Fz (*t*(340.94) = 2.93, *p* = 0.003) and Cz (*t*(335.55) = 8.28, *p* < 0.001), whereas no difference was found at Pz (*p* = 0.31).

The interaction between Feedback Condition and Channel (Fig. 6B) revealed that only at the parietal electrode Pz, emotional feedback led to a significant larger amplitude than non-emotional feedback *t*(171) = 6.35, *p* < 0.001), whereas this was not the case for Cz (*p* = 0.18) and Fz (*p* = 0.68). Also, P3b amplitude after emotional feedback was larger at Pz vs. Cz (*t*(171) = − 4.05, *p* < 0.001) and Cz vs. Fz (*t*(171) = 9.23, *p* < 0.001). After non-emotional feedback, the mean P3b amplitude was larger at Cz vs. Fz (*t*(171) = 7.98, *p* < 0.001), whereas no difference was found for Cz vs. Pz (*p* = 0.229).

Resolving the interaction between Valence and Channel (Fig. 6C) revealed that negative feedback elicited a smaller mean P3b amplitude than positive feedback at channel Cz (*t*(171) = − 3.48, *p* < 0.001), whereas no such differences were found at frontal or parietal areas (all *p* values > 0.17). After negative feedback, the mean P3b amplitude was significantly larger at Pz vs. Cz (*t*(171) = − 3.63, *p* = 0.001) and Cz vs. Fz (*t*(171) = 6.03, *p* < 0.001), in contrast to positive feedback, for which there was a larger P3b amplitude at Cz vs. Fz (*t*(171) = 12.1, *p* < 0.001), but no significant amplitude differences for Cz vs. Pz (*p* = 0.068).

**Fig. 7:**
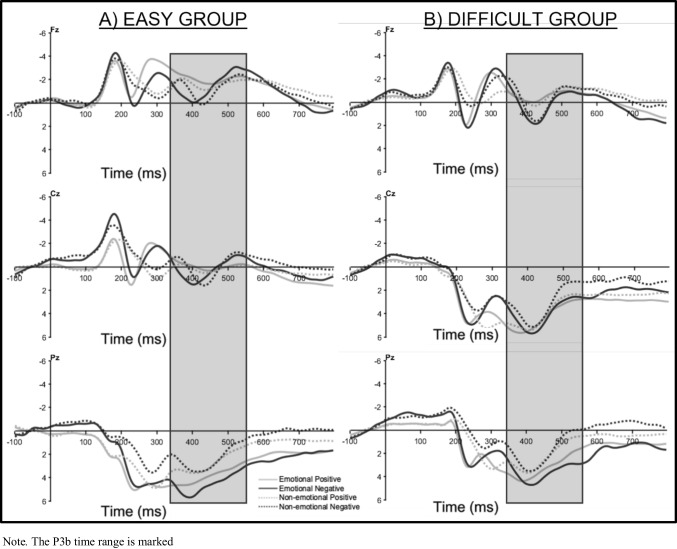
Feedback-locked ERP waveforms for the easy (**A**) and difficult (**B**) groups across three midline electrodes: Fz, Cz and Pz

No main effect of Task Difficulty (*p* = 0.078) nor an interaction between Task Difficulty and Feedback Condition (*p* = 0.327) was found. Figure 7 illustrates the mean P3b waveforms.

## Discussion

We used a probabilistic learning task to investigate whether young adults’ learning performance would benefit from emotional feedback during conditions with high working memory load. Additionally, we examined whether improved learning would be accompanied by improved detection of unexpected events and improved working memory updating following emotional feedback.

### Learning performance

Learning performance increased over the course of the experiment as reflected by increasing accuracy and decreasing reaction times. As expected, learning was worse in the difficult compared with the easy task, indexed by lower accuracy and longer reaction times. As for the effect of emotional feedback, we found that accuracy was higher in the condition with emotional than non-emotional feedback for both groups. This leads us to suggest that different from our expectations, younger adults benefitted from emotional feedback under both working memory load conditions. This finding is in line with results of Hurlemann et al. (2010), where younger adults’ learning performance was better after social feedback in a comparable task. This is partly explained by the social facilitation theory, which states that in well-learned tasks social stimuli may facilitate performance, while they inhibit performance in more demanding tasks (Geen & Gange, 1977). However, this would possibly have led to a disadvantage of emotional feedback under high working memory load, which was not the case in our study. An alternative explanation could be the motivational relevance of the shown social stimuli and the direct comparison between emotional and non-emotional feedback in our study (Pfabigan & Han, 2019; Sailer et al., 2023).

Our findings differ from a recent study by Ferdinand and Hilz (2020), who found no advantage of emotional feedback in an easy probabilistic learning task for younger adults. This discrepancy may be attributed to the use of neutral faces as feedback stimuli in their study, which potentially still elicited emotional (surprise) responses. Nonetheless, they demonstrated the benefits of emotional feedback for older individuals. One possible interpretation is that, unlike in older adults, where emotional feedback improves processing and compensates for age-related impairments, younger adults may require cognitive resources for processing additional emotional information. This is supported by our reaction time data: Under low working memory load, participants benefitted from emotional feedback early in the experiment, while this advantage emerged later, after they had reached a certain level of proficiency, under high working memory load.

### Feedback-related negativity

We expected a greater advantage of emotional feedback under high working memory load; thus, we anticipated an increased peak-to-peak FRN in response to emotional compared with non-emotional feedback, particularly in the difficult group. However, we did not find an interaction between feedback condition and task difficulty and no difficulty effect on the unsigned reward prediction error, which is reflected in the peak-to-peak FRN. These results are in contrast to Krigolson et al. (2015), who found a reduced detection of unexpected events during a condition with high working memory load, by adding a second task to a time estimation task (by telling participants that their eye movement will be recorded and that they would be evaluated on how well they performed). This secondary task, however, might have had an effect not so much on working memory load, but instead might have added motivational significance to the task (Weinberg & Hajcak, 2010). In contrast, our study was designed to increase working memory load by increasing the number of stimulus–response associations, which worked, according to the behavioural difficulty effects. Nevertheless, we could not find an effect of this manipulation on the peak-to-peak FRN amplitude, which corresponds to recent literature, according to which the FRN seems to be a rapid evaluation process that is not interfered with by higher working memory demands (Arbel, 2020; Ferdinand, 2019; Somon et al., 2019).

Conversely, we found that emotional stimuli elicited enhanced feedback processing, as indicated by the fact that both groups, regardless of working memory load, demonstrated an increased FRN after emotional compared with non-emotional information. This aligns with previous studies that involved social stimuli, underscoring an augmented detection of expectancy violations in socioemotional contexts (Pfabigan & Han, 2019; Pfabigan et al., 2019; Schulreich et al., 2013). This heightened responsiveness may be attributed to the motivational significance and salience of social stimuli at a fundamental level (Weinberg & Hajcak, 2010). Motivational significance may even have been emphasized by the use of face feedback and the explicit mention that the facial expression would provide a performance evaluation. Our results differ from those of Ferdinand and Hilz (2020), who observed an enlarged FRN after non-emotional feedback compared with emotional feedback. Yet, it is crucial to note that their use of neutral faces in the non-emotional condition could have evoked larger expectancy violations due to their uncommonness and possibly negative connotation in everyday communication. With the inclusion of a non-emotional condition, which we tried to develop as similarly complex as the face stimuli, we also saw this benefit in younger adults.

Additionally, we identified that negative feedback elicited a larger peak-to-peak FRN than positive feedback for both groups and both feedback conditions. This aligns with common observations that negative feedback generates a larger FRN, explained by less expected negative feedback after having learned (Ferdinand, 2019; Ferdinand & Hilz, 2020; Holroyd & Coles, 2002; Pfabigan et al., 2019; Schulreich et al., 2013).

Taken together, our findings indicate that the unsigned reward prediction error is not inherently sensitive to working memory load; rather, it is predominantly influenced by emotional information, which is likely due to the salience of the feedback received and the common observation that the FRN is generated by less expected (here negative) feedback when learning has taken place (Holroyd & Coles, 2002).

### P3b

In our study, we additionally investigated working memory updating as reflected in the mean P3b amplitude. We hypothesized that especially under high working memory load, emotional feedback would lead to an enhanced working memory updating.

Contrary to our expectations, we did not find an interaction between the emotionality of the feedback and higher working memory load. As for emotionality, we found a larger mean P3b amplitude at posterior electrode sites for emotional feedback (Martín, 2012; Polich, 2007). This result suggests that emotional feedback has a distinct impact on the neural processing of feedback by strengthening working memory updating processes, which are typically associated with the parietal P3b (Martín, 2012; Pfabigan et al., 2014, 2019; Polich, 2007; Schulreich et al., 2013). However, these results contrast the ones from Ferdinand and Hilz (2020), who could not find a benefit in working memory updating after emotional feedback in younger adults in a task that was similar to the easy condition. A possible explanation could be a ceiling effect in the younger adults’ sample in the previous study, which could have prevented an effect of emotionality to become visible.

For task difficulty, we found that it did not change the size, but rather the topography of the P3b. Whereas the P3b showed a clear parietal maximum under low working memory load, it displayed a broader distribution, i.e., a parieto-central distribution under high working memory load. These different P3b topographies between the easy and difficult condition might suggest that task difficulty, here induced by manipulating working memory load, influences the neural mechanisms underlying attentional resource allocation during working memory updating. The parietal P3b distribution observed in the easy group aligns with the characteristic topography typically found in tasks with moderate attentional demands, where parietal regions are engaged in context updating and stimulus evaluation processes (Ferdinand, 2019; Ferdinand & Hilz, 2020; Polich, 2007). This suggests that the P3b component plays a role in integrating incoming information into the existing cognitive context and points to an efficient allocation of cognitive resources for the evaluation of feedback and the adjustment of behavior (Wurm et al., 2020). In contrast, the shift to a central P3b maximum in the difficult condition indicates an additional engagement of more central and frontal regions, which are usually associated with compensatory enhanced cognitive control processes (Ferdinand, 2019; Reuter-Lorenz et al., 2000; Segalowitz et al., 2001; Tregellas et al., 2006).

The absence of a significant interaction between task difficulty and feedback condition suggests that the effects of higher working memory load and feedback condition on the P3b amplitude are largely independent. Emotional feedback leads to an enhanced working memory updating independently of working memory load. In contrast, a higher working memory load as induced by a higher task difficulty does not result in enhanced working memory updating per se, but to an additional engagement of frontocentral networks becoming more engaged in difficult tasks to support increased cognitive control and attentional demands.

A third effect was that positive feedback elicited a larger P3b than negative feedback at central electrode sites. This might suggest that positive feedback has been harder to process and thus more frontal resources were needed for it to be processed adequately (Ferdinand, 2019; Reuter-Lorenz et al., 2000; Segalowitz et al., 2001; Tregellas et al., 2006). A hint towards this interpretation can be found in our exploratory analyses, including Learning Half as a factor (see Supplementary Material).We found that in the easy group, P3b amplitude at central and parietal electrode sites significantly decreased from the first to the second learning half for negative feedback, while this was not the case for positive feedback. This could indicate that negative feedback was not as difficult to process and probably not as informative after learning had taken place (Eppinger et al., 2008; Holroyd & Coles, 2002). However, these results should be interpreted with caution because of a low statistical power.

### Limitations

We selected a between-subjects design due to the large number of trials necessary for reliable ERP analyses to avoid participant exhaustion and practice effects that might have occurred in a within-subjects design. By restricting each participant to one condition, we sought to minimize these problems. However, we acknowledge that a between-subjects design has certain limitations, such as possible variability between groups and the need for larger sample sizes to achieve a statistical power comparable to that of within-subject designs. Despite these challenges, this approach was considered the most appropriate one for the study’s objectives. Additionally, we included some measures to control for possible baseline differences in learning ability and measures of crystallized and fluid intelligence between participant groups.

An influential question in EEG research investigating feedback processing is the use of comparably complex feedback stimuli to rule out possible differences in feedback perception (Liu & Gehring, 2009; Pfabigan et al., 2019). In the present study, we tried to reach this goal by contrasting emotional faces with scrambled images of neutral faces from the same database. Our intention was to create non-emotional stimuli that still share critical aspects with faces while eliminating emotional information. However, the scrambled images could still have been perceived as unusual and therefore might have influenced our results. Nevertheless, compared with Ferdinand and Hilz (2020), who used neutral face stimuli in the non-emotional condition, we obtained overall much smaller peak-to-peak FRNs in the non-emotional condition, which means that the present stimuli elicited smaller expectancy violations than neutral faces. This speaks in favor of our stimulus choice. Either way, future research should strive to create non-emotional stimuli that are even more comparable to emotional faces on all possible dimensions but emotionality. This idea can be supported by the ERP waveforms (especially in the easy group), in which non-emotional feedback elicited a delayed FRN. This could be due to the less effective discriminability of the scrambled faces, which we actually aimed to prevent by adding intuitive colours to emotional and non-emotional feedback. Faces are perceived as especially complex, but maybe the scrambled faces were perceived as even more complex stimuli, so participants needed more time to process them adequately. Future research therefore could try to manipulate the emotionality of the feedback stimuli by morphing images to a greater vs. lesser extent. By this, one could avoid additional colouring of the faces to convey the valence of the feedback. Our choice to do use colour-coded feedback introduced an additional perceptual cue in the emotional feedback condition, rendering the emotion of the faces irrelevant. Nevertheless, significant effects of emotionality were still observed, suggesting that the presence of emotional content may have engaged additional cognitive processing and supported learning.

In future research, a detailed investigation of the learning process over time and its possible modulation by emotional feedback would be interesting. This could be achieved through the joint modeling of behavioral and EEG data, which was beyond the scope of the present study. Also, because of the limited number of included trials (please see supplementary material), it was not possible to segment the EEG data into the same four bins as the behavioral data. For example, implementing a learning paradigm with a lower proportion of valid stimulus–response associations may help to overcome these issues in future studies. With this approach, enough negative feedback trials could be included to perform a precise modeling approach to investigate the learning process.

Another concern regarding our FRN results that should be addressed is that of contamination by possible component overlap. More specifically, in the ERP waveforms right after feedback onset, a difference between positive and negative feedback is visible in the time range between 50 and 100 ms. Although an interval of 500 ms between response and stimulus presentation is not uncommon for learning paradigms (Arbel & Fox, 2021; Arbel et al., 2017; Bellebaum & Daum, 2008; Eppinger et al., 2008, 2009; Ferdinand, 2019; Ferdinand & Hilz, 2020; Frank et al., 2005; Herbert et al., 2011; Weismüller & Bellebaum, 2016), we cannot exclude that this difference might reflect residual response-locked activity. It should be noted that the peak-to-peak quantification of the FRN that we used in our analyses should be robust to baseline differences and other differences in the ERP that occur before the P2, because this method of quantification uses the P2 peak as a baseline to measure the following N2 peak (Handy, 2005; Picton et al., 2000). Because this difference appears to not be present in the P2 time range any more, we think that the effects we found should still be reliable. Nevertheless, using a deconvolution method to reduce the influence of possibly overlapping processing steps could be helpful for future studies (Ehinger & Dimigen, 2019).

## Conclusions

Our study was designed to investigate whether emotional feedback could reduce learning deficiencies associated with higher working memory load in younger adults. Our findings revealed that during both working memory load conditions, younger adults showed improved performance after emotional feedback, suggesting that emotional feedback can support reinforcement learning. Additionally, the neural processes involved in feedback processing, the detection of unexpected events (as reflected in the peak-to-peak FRN) and working memory updating (as measured with the P3b) were strengthened by emotional feedback. A high working memory load led to decreased learning performance and supposedly the need to recruit more cognitive resources during working memory updating. However, the detection of unexpected events remained unaffected by increased working memory load. The unexpected lack of an interaction between emotionality and working memory load on the FRN and P3b suggests that these factors independently influence feedback processing.

## Author´s contributions

JI Braunwarth: Conceptualization, Data curation, Formal Analysis, Investigation, Methodology, Software, Validation, Visualization, Writing: Original Draft Preparation, Writing: Review & Editing.

NK Ferdinand: Conceptualization, Funding Acquisition, Methodology, Project Administration, Resources, Validation Writing: Review & Editing, Supervision.

## Supplementary Information

Below is the link to the electronic supplementary material.

Supplementary file1 (PDF 318 kb)

## Data Availability

The data underlying this article will be shared upon request.

## Code availability

The code underlying this article will be shared upon request.
