# Supplementary material for: The impact of emotional feedback in learning easy and difficult tasks – an ERP study
Source: Cogn Affect Behav Neurosci. 2025 Mar 27;25(4):971–88. doi: 10.3758/s13415-025-01284-2 (PMC12356731; doi:10.3758/s13415-025-01284-2)
Supplement: Supplementary file 1 — (PDF 318 kb) [file 13415_2025_1284_MOESM1_ESM.pdf]

Supplementary Material

The Impact of Emotional Feedback in Learning Easy and Difficult Tasks – An ERP study

Jana I. Braunwarth, Nicola K. Ferdinand

Table S1

Mean number (Standard Deviations) of included trials for Group x Learning Half x Feedback Condition x Valence.

Variable	Easy Group		Difficult Group	
	1st Learning Half	2nd Learning Half	1st Learning Half	2nd Learning Half
Emotional correct	127.90 (12.05)	145.23 (8.38)	102.00 (16.79)	104.57 (14.02)
Emotional incorrect	27.82 (8.47)	10.95 (4.97)	45.86 (13.35)	49.67 (14.67)
Non-emotional correct	118.36 (13.09)	139.50 (9.41)	126.24 (16.59)	123.33 (18.27)
Non-emotional incorrect	32.82 (9.97)	16.82 (8.61)	29.81 (19.36)	29.24 (14.68)

Exploratory ERP Analyses

In this exploratory analysis, we were interested in investigating whether learning half has an impact on the feedback-locked ERPs. Due to the low trial numbers in certain conditions (see table S1), the following analyses are likely not reliable and should be interpreted with caution.

Peak-to-Peak FRN

We conducted an exploratory peak-to-peak FRN ANOVA at channel FCz, incorporating the between-subject factor Task Difficulty (easy, difficult) and the within-subject factors Feedback Condition (emotional, non-emotional), Valence (positive, negative), and Learning Half (first, second). Significant main effects and interactions are reported in Table S2, and a graphical overview is provided in Figure S1. Including Learning Half as a factor did not yield any additional significant effects or interactions (all p-values >.15).

Table S2

Effects of the Peak-to-peak FRN ANOVA.

Measure	DFn	DFd	F-value	p-value	p<.05	η_p^2
Difficulty	1	41	3.67	0.06		0.08
**Feedback Condition**	**1**	**41**	**62.64**	**0.00000000087**	*****	**0.60**
**Valence**	**1**	**41**	**25.82**	**0.00000862000**	*****	**0.38**
Learning Half	1	41	0.19	0.66		0.01
Difficulty x Feedback Condition	1	41	1.52	0.22		0.04
Difficulty x Valence	1	41	2.24	0.14		0.05
Difficulty x Learning Half	1	41	0.17	0.68		0.00
Feedback Condition x Valence	1	41	0.09	0.76		0.00
Feedback Condition x Learning Half	1	41	1.69	0.20		0.04
Valence x Learning Half	1	41	2.17	0.15		0.05
Difficulty x Feedback Condition x Valence	1	41	0.97	0.33		0.02
Difficulty x Feedback Condition x Learning Half	1	41	0.01	0.91		0.00
Difficulty x Valence x Learning Half	1	41	0.03	0.86		0.00
Feedback Condition x Valence x Learning Half	1	41	2.13	0.15		0.05
Difficulty x Feedback Condition x Valence x Learning Half	1	41	0.89	0.36		0.02

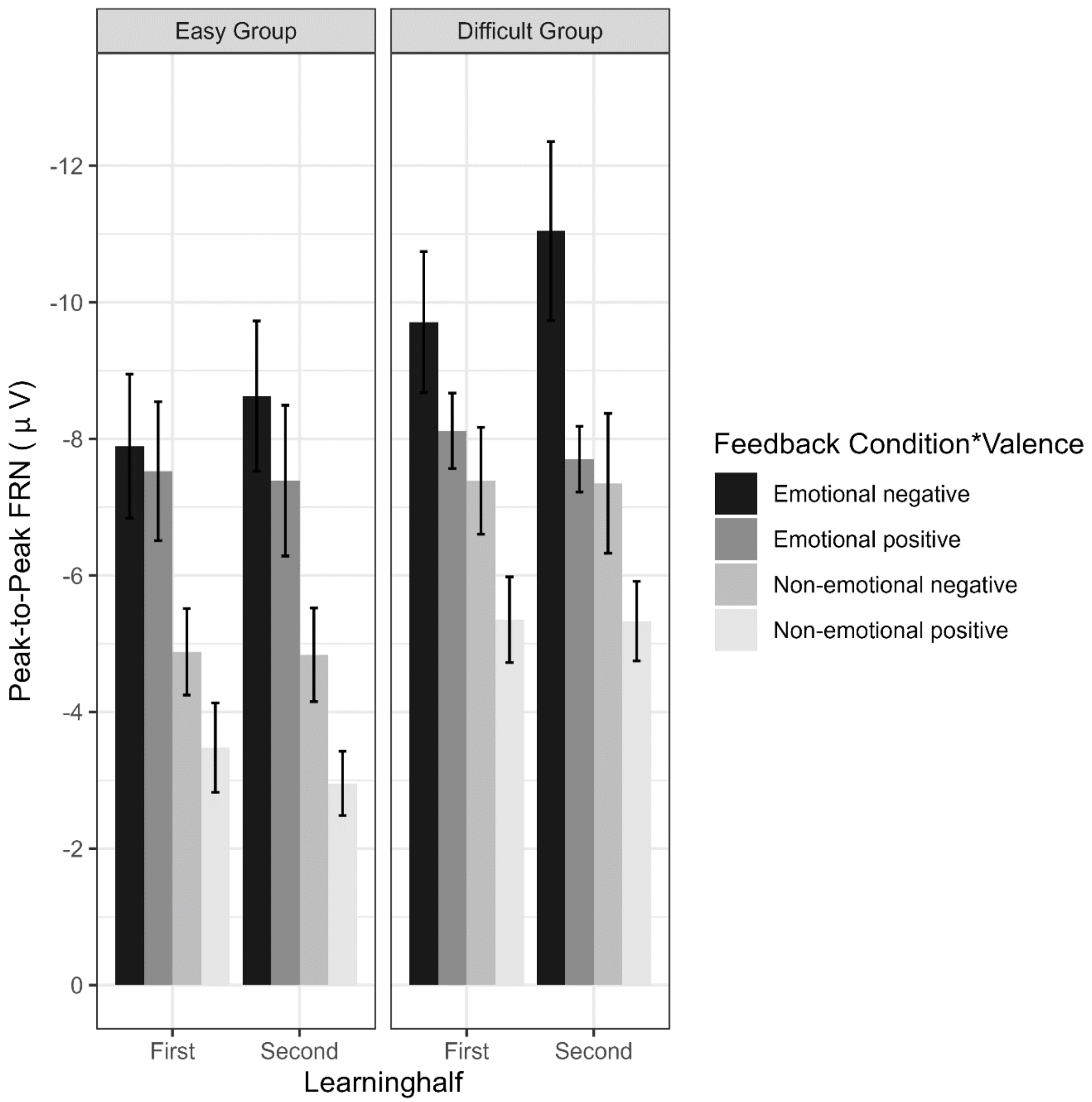

Figure S1. *Graphical illustration for the exploratory peak-to-peak FRN analyses*.

Mean P3b

We performed an exploratory mean P3b ANOVA with the between-subject factors Task Difficulty (easy, difficult) and the within-subject factors Feedback Condition (emotional, non-emotional), Valence (positive, negative), Learning Half (first, second) and Channel (Fz, Cz, Pz). The significant main effects and interactions can be found in table S3. Notably, no interactions of the factor Learning Half were found with Feedback Condition. Also, no interactions including Feedback Condition x Task Difficulty emerged. However, Learning Half did interact with Task Difficulty.

Table S3

Results of the ANOVA on the mean P3b amplitude.

Measure	DFn	DFd	F-value	p-value	p<.05	η_p^2
Difficulty	1	41	3.27	0.08		0.07
Valence	1	41	1.14	0.29		0.03
**Feedback Condition**	**1**	**41**	**5.39**	**0.025**	*****	**0.12**
**Channel**	**1.55**	**63.63**	**22.19**	**0.000000507**	*****	**0.35**
**Learning Half**	**1**	**41**	**23.36**	**0.0000191**	*****	**0.36**
Difficulty x Valence	1	41	0.01	0.94		0.00
Difficulty x Feedback Condition	1	41	0.98	0.33		0.02
Difficulty x Feedback Condition	1	41	0.98	0.33		0.02
**Difficulty x Channel**	**1.55**	**63.63**	**7.39**	**0.003**	*****	**0.15**
Difficulty x Learning Half	1	41	1.69	0.20		0.04
Valence x Feedback Condition	1	41	2.04	0.16		0.05
**Valence x Channel**	**2**	**82**	**5.58**	**0.005**	*****	**0.12**
**Feedback Condition x Channel**	**1.72**	**70.39**	**11.45**	**0.000115**	*****	**0.22**
**Valence x Learning Half**	**1**	**41**	**5.02**	**0.031**	*****	**0.11**
Feedback Condition x Learning Half	1	41	0.06	0.82		0.00
**Channel x Learning Half**	**2**	**82**	**22.39**	**0.0000000175**	*****	**0.35**
Difficulty x Valence x Feedback Condition	1	41	0.33	0.57		0.01
Difficulty x Valence x Channel	2	82	0.01	0.98		0.00
Difficulty x Feedback Condition x Channel	1.72	70.39	0.97	0.37		0.02
**Difficulty x Valence x Learning Half**	**1**	**41**	**5.17**	**0.028**	*****	**0.11**

Measure	DFn	DFd	F-value	p-value	p<.05	η_p^2
Difficulty x Feedback Condition x Learning Half	1	41	0.65	0.43		0.02
**Difficulty x Channel x Learning Half**	**2**	**82**	**3.19**	**0.046**	*****	**0.07**
Valence x Feedback Condition x Channel	1.56	64.11	0.94	0.37		0.02
Valence x Feedback Condition x Learning Half	1	41	1.19	0.28		0.03
**Valence x Channel x Learning Half**	**2**	**82**	**6.34**	**0.003**	*****	**0.13**
Feedback Condition x Channel x Learning Half	2	82	2.47	0.09		0.06
Difficulty x Valence x Feedback Condition x Channel	1.56	64.11	0.21	0.76		0.01
Difficulty x Valence x Feedback Condition x Learning Half	1	41	1.89	0.18		0.04
**Difficulty x Valence x Channel x Learning Half**	**2**	**82**	**3.57**	**0.033**	*****	**0.08**
Difficulty x Feedback Condition x Channel x Learning Half	2	82	1.12	0.33		0.03
Valence x Feedback Condition x Channel x Learning Half	1.58	64.64	0.49	0.57		0.01
Difficulty x Valence x Feedback Condition x Channel x Learning Half	1.58	64.64	0.83	0.42		0.02

To explain the significant four-way interaction between Difficulty, Valence, Channel, and Learning Half, we conducted separate ANOVAs for each difficulty condition. The analysis for the easy group revealed significant main effects for Channel ($F(2,42) = 19.74$, $p < .001$, $\eta_p^2 = .48$),

Learning Half ($F(1,21) = 14.36$, $p = .001$, $\eta_p^2 = .41$), a two-way interaction between Valence and Learning Half ($F(1,21) = 9.47$, $p = .006$, $\eta_p^2 = .31$), and a three-way interaction between Valence and Channel and Learning Half ($F(2,42) = 6.94$, $p = .002$, $\eta_p^2 = .25$). To resolve the three-way interaction between Valence, Channel, and Learning Half in the easy group, we conducted Bonferroni-corrected Post hoc tests. These showed a clear parietal distribution of the P3b in Learning Half 1 (positive feedback: Fz vs. Cz: $t(85.99) = -3.59$, $p = .002$, and Cz vs. Pz: $t(85.83) = -4.57$, $p < .001$, negative Feedback: Fz vs. Cz: $t(85.80) = -2.74$, $p = .022$, and Cz vs. Pz: $t(85.90) = -3.07$, $p = .009$), which became even more focused on parietal electrode sites in Learning Half 2 (positive feedback: Fz vs. Cz: $p = .149$, and Cz vs. Pz: $t(85.92) = -4.91$, $p < .001$, negative Feedback: Fz vs. Cz: $p = .948$, and Cz vs. Pz: $t(85.68) = -4.37$, $p < .001$). Differences between the first and the second half were significant, however, only for negative feedback at electrodes Cz ($t(85.85) = 4.53$, $p < .001$) and Pz ($t(84.49) = 2.84$, $p = .005$). Together, these effects may suggest a learning effect, indicating that working memory updating requires fewer frontal resources as learning progresses, with this reduction being particularly pronounced in response to negative feedback.

The analysis for the difficult group revealed significant main effects for Channel ($F(2,40) = 10.86$, $p < .001$, $\eta_p^2 = .35$), and Learning Half ($F(1,20) = 9.51$, $p = .006$, $\eta_p^2 = .32$), and a significant interaction between Channel and Learning Half ($F(2,40) = 8.68$, $p < .001$, $\eta_p^2 = .30$).

To explain the two-way interaction between Channel and Learning Half in the difficult condition, we also calculated Bonferroni-corrected Post hoc tests. These tests revealed a centro-parietal distribution of the P3b in the first and the second learning half (Learning Half 1: Fz vs. Cz: $t(83) = -10.04$, $p < .001$), and Cz vs. Pz: $p = .245$, Learning Half 2: Fz vs. Cz: $t(83) = -8.04$, $p < .001$), and Cz vs. Pz: $p = .278$). Differences between the first and the second half were significant, however, only for electrodes Cz ($t(83) = 4.98$, $p < .001$) and Pz ($t(83) = 4.05$, $p < .001$), whereas no significant differences were found at frontal sites (Fz: $p = .152$). Please see figure S2 for a graphical illustration. All other results (not including the factor Learning Half) are detailed in the main manuscript.

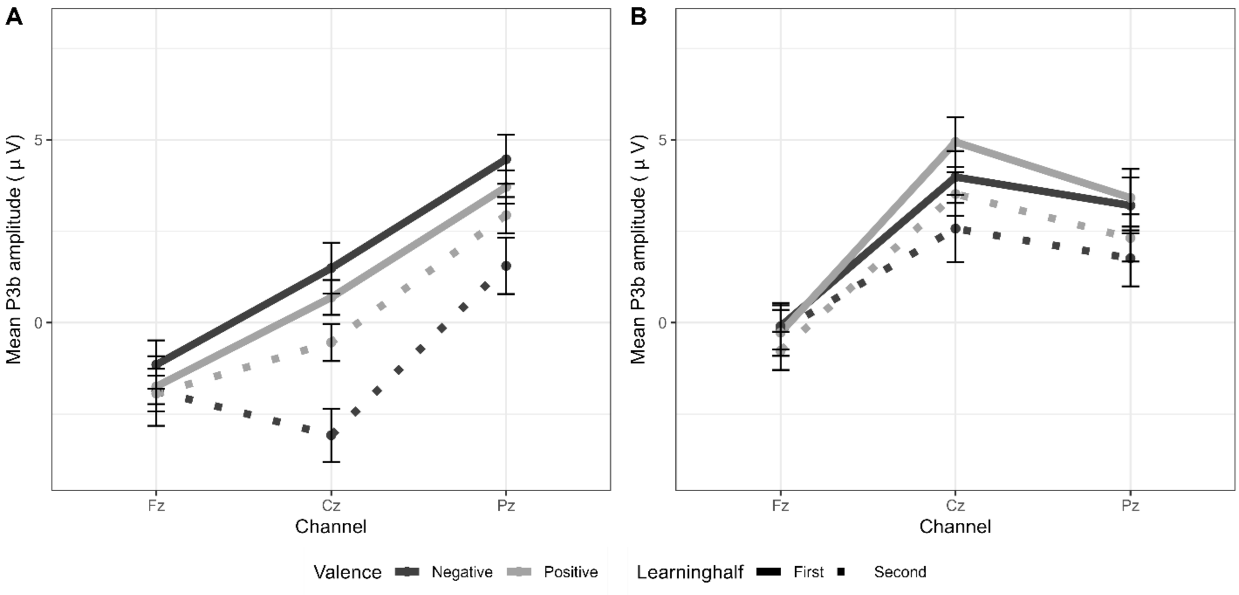

Figure S2. *Graphical illustration for the exploratory mean P3b analysis resolving the four-way interaction for A) the easy and b) the difficult group.*
